# The preparedness and knowledge of pharmacists and general practitioners in managing human monkeypox: a highly spreading infectious disease

**DOI:** 10.1186/s40545-023-00636-y

**Published:** 2023-10-23

**Authors:** Asmaa A. Elsayed, Hoda M. Rabea, Salman Ahmed Salman, Engy A. Wahsh

**Affiliations:** 1 Clinical Pharmacy Department, Faculty of Pharmacy, Sohag University, Sohag, Egypt; 2 Clinical Pharmacy Department, Faculty of Pharmacy, Beni-Suef University, Beni Suef, Egypt; 3 Faculty of Pharmacy, Sohag University, Sohag, Egypt; 4 Clinical Pharmacy Department, Faculty of Pharmacy, October 6 University, Giza, Egypt

**Keywords:** Pharmacist, Monkeypox, Epidemic, Questionnaire, Awareness

## Abstract

**Background:**

After the era of the COVID-19 pandemic, the role of pharmacists was emphasized in the battle against highly spreading and infectious diseases like human Monkeypox (hMPV).

**Aim:**

Assess the hMPV knowledge of the community, clinical pharmacists, and general practitioners (GPs) and raise their awareness about hMPV.

**Methods:**

A web-based questionnaire was distributed randomly to Egyptian community pharmacists, clinical pharmacists, and GPs from all governorates. The questionnaire was divided into two sections: one for demographic information and the other for hMPV knowledge (nature of the disease, incubation period, transmission, symptoms, Prophylaxis, Prevention, and management). The evidence-based answers were provided after completing the submission. Data were descriptively analyzed using IBM SPSS software.

**Results:**

From a total of 753 respondents, only 710 participants were included in the final data analysis. The % of respondents who presented good total knowledge scores about hMPV was comparable between study groups (*P =* 0.826). There were no differences between groups identifying different disease clinical characteristics (*P =* 0.689) and hMPV management (*P =* 0.324). Community pharmacists had better knowledge scores than GPs in the prevention and prophylaxis domain (*P =* 0.037).

**Conclusion:**

Pharmacists and GPs have good and similar knowledge levels of hMPV. However, a gap exists in recognizing the right hMPV incubation period, prophylaxis, and omitting antibiotics from hMPV management. Pharmacists and GPs are the frontline health care providers (HCPs), so they would require more knowledge enhancement about such contagious diseases to offer the best possible patient care.

## Introduction

Human Monkeypox Virus (hMPV) is a double-stranded DNA virus transmitted to humans and belongs to the Poxviridae family, including the smallpox virus [1].

The incidence of hMPV has escalated dramatically after the first case was discovered in the United Kingdom on May 6, 2022. In October 2022, approximately 71,096 cases were identified globally, and the Egyptian Ministry of Health and Population (MOHP) confirmed the first case of hMPV in September 2022 [2, 3].

hMPV symptoms range from mild to severe, with itchy and painful skin rash, as well as fever, diffuse headache, lethargy, lymphadenopathy, and myalgia [4, 5]. Skin rash can proceed from papules to vesicles, pustules, and crusts on the face, torso, and extremities [6].

Reporting of hMPV complications include secondary bacterial infection, Sepsis, encephalitis, dehydration, and Pneumonia. WHO reported a raised fatality rate, especially among children, with 26 deaths attributed to the 2022 hMPV outbreak [2, 7].

Antivirals are offered for treatment, and smallpox vaccines are recommended as a preventive measure. The crucial role of health care providers (HCPs) in disease control is to improve preventative measures through active surveillance and optimized diagnosis and treatment [7]. Past studies concerning hMPV knowledge neglected the role of pharmacists in the current outbreak [7–9].

The abrupt and unexpected increase in the incidence of hMPV in multiple non-endemic countries implies the need to raise awareness about the present outbreak to minimize undetected disease transmission [2, 10].

In Egypt, medical practices present pharmacists and general practitioners (GP) as the frontline HCPs serving the public in pharmacies and hospitals [11]. The role of pharmacists in the COVID-19 pandemic was emphasized, especially in counseling, minimizing misinformation, and raising awareness among the public [12]. Also, Egyptian pharmacists and GPs exerted a primary role in administering COVID-19 vaccines as a part of the Egyptian MOHP national campaign [13].

Consequently, evaluating Egyptian pharmacists and GP knowledge could reflect the current confidence levels in diagnosing and managing hMPV-infected cases. Moreover, we aimed to address knowledge gaps regarding hMPV and provided evidence-based answers to equip them with up-to-date information to enhance their engagement with the public.

## Methods

### Study design

A cross-sectional questionnaire targeting actively working pharmacists and GPs was conducted using a web-based questionnaire. Data were gathered from Sep 15, 2022, to Oct 15, 2022.

### Data collection tool

The development of this questionnaire was adapted from a previously published study [7] and modified based on the existing literature, especially the WHO and the Centers for Disease Control and Prevention (CDC) [2, 3]. Minor modifications were made to develop the final version by deleting and merging questions. The final version was provided in Arabic and English and included 20 questions divided into two sections. The first section consisted of seven questions about the participant's demographics (Age, gender, occupation, and residence). The other section contained 13 questions about hMPV knowledge (nature of the disease, statistics, disease transmission, symptoms, management, preventive measures, and vaccination). Questions varied between open-ended and closed-ended questions.

After finishing the questionnaire, each participant will have a score and be provided evidence-based answers to raise their awareness and knowledge about hMPV. The calculated Cronbach's alpha to test the internal consistency of the questionnaire items was 0.741.

### Participant selection

The questionnaire was randomly and anonymously distributed by private E-mails (contacts of authors or recruited from hospital registries) and social media (Facebook groups, Messenger, and WhatsApp) with a follow-up reminder after the initial message within 1 week.

This study included actively working pharmacists (community and clinical pharmacists) and GPs aged above 20 years. Physicians with a postgraduate degree, pharmacists in pharmaceutical advertisement, and pharmacy/medicine undergraduate students were all excluded.

### Sample size calculation

For sample size calculations, the targeted population was about 200,000 working pharmacists and GPs in Egypt, with a confidence level of 95% and a margin of error of 5%. Assuming that 50% of the pharmacists and GPs would have good knowledge about hMPV, the minimum acceptable sample size is 507 participants.

### Statistical analysis

IBM SPSS statistical package version 26 was used for data management and analysis. Continuous variables were expressed as numbers and percentages, while the numerical data were expressed as mean and standard deviation (SD). The differences in hMPV knowledge between pharmacists and GPs were assessed using the Chi-square test (*P* values ≤ 0.05 were considered statistically significant). The One-way ANOVA test and independent samples *T*-test were used to test the differences between numerical variables and the Chi-square tests for categorical variables. Pearson Correlation was used to determine the impact of medical education on different hMPV-related Knowledge domains.

## Results

**Fig. 1:**
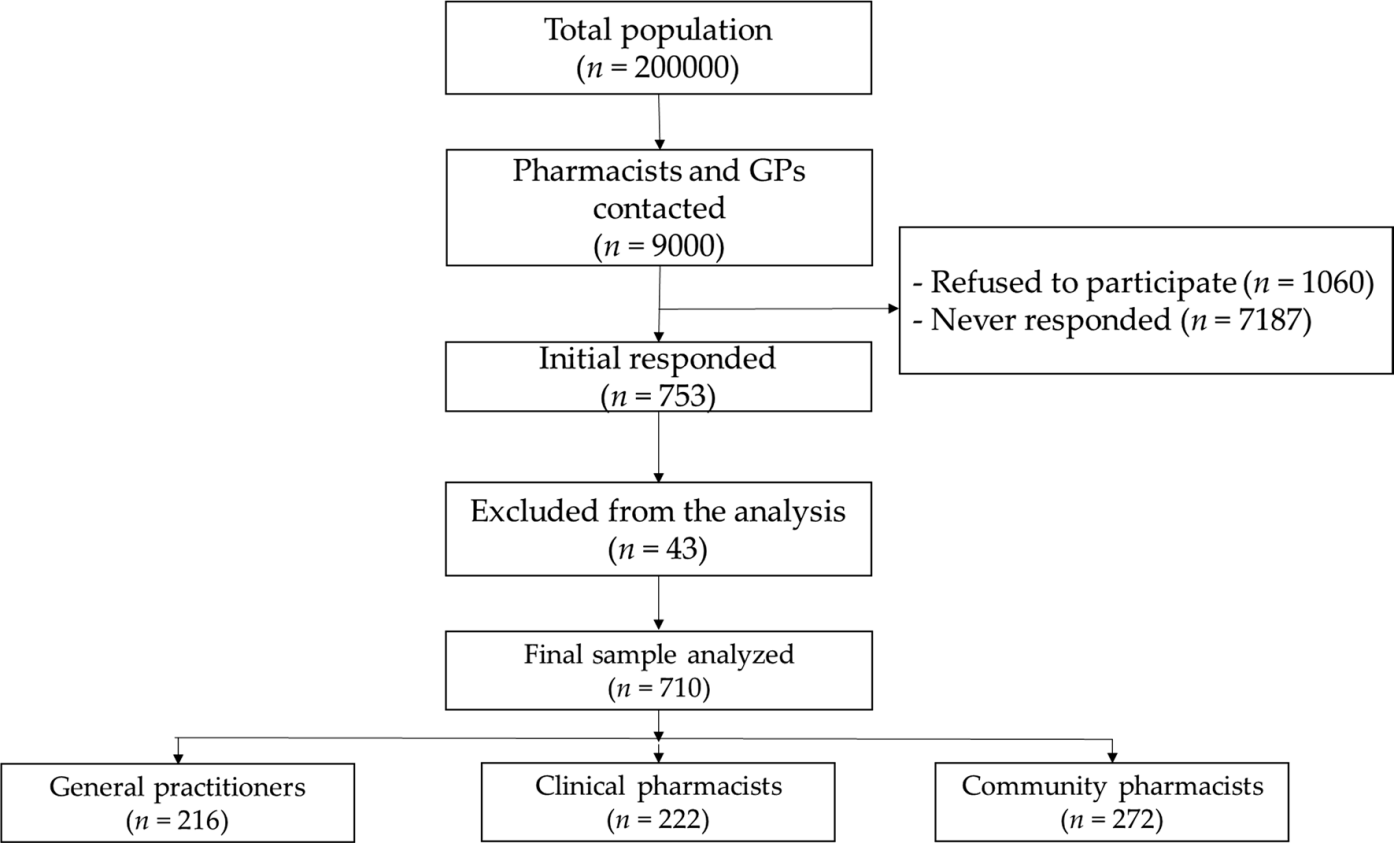
Study recruitment flow chart

Of the 753 participants who initially responded to the survey, 43 did not meet our inclusion criteria and were excluded from the data analysis. A total of 710 pharmacists and GPs completed the survey [272 (38.2%) community pharmacists, 216 (30.6%) GPs, and 222 (31.2%) clinical pharmacists] (Fig. 1 illustrates the recruitment process). The participants were 288 (40.6%) males and 422 (59.4%) females who ranged in age from 21 to 58 years (demographic data are summarized in Table 1).

**Table 1:** Demographic data of study participants

Variables	Community pharmacists	Clinical pharmacists	General practitioners	P-value
Gender				
Male	106 (39.0%)	89 (40.1%)	103 (47.7%)	0.051
Female	166 (61.0%)	133 (59.9%)	113 (52.3%)	
Age				
Range	21–57	21–55	23–49	0.0001
Mean ± SD	26.15 ± 5.54	28.60 ± 6.73	27.12 ± 4.89	
Residence				
Upper Egypt	97 (35.7%)	89 (40.0%)	86 (39.8%)	0.773
Center	92 (33.8%)	69 (31.0%)	63 (29.2%)	
Delta	83 (30.5%)	64 (29.0%)	67 (31.0%)	
Recent medical education on hMPV				
Yes	54 (20.0%)	51 (23.0%)	52 (24.1%)	0.659
No	218 (80.0%)	171 (77.0%)	164 (75.9%)	
Egypt reported positive cases				
Yes	111 (40.8%)	107 (48.2%)	109 (50.5%)	0.045
No	49 (18.0%)	29 (13.1%)	20 (9.3%)	
I do not know	112 (41.2%)	86 (38.7%)	87 (39.2%)	
Positive cases in nearby countries				
Yes	115 (42.3%)	111 (50.0%)	120 (55.6%)	0.061
No	25 (9.2%)	16 (7.2%)	13 (6.0%)	
I do not know	132 (48.5%)	95 (42.8%)	83 (38.4%)	
Total knowledge scores				
Range	4–23	5–24	4–23	0.793
Mean ± SD	12.67 ± 3.48	12.86 ± 3.22	12.68 ± 3.20	

Statistically significant (*p* < 0.05)

Data are reported as numbers (Percentages) and mean ± standard deviation (SD)

Pharmacists and GPs reported similarly receiving recent medical education about hMPV (*P =* 0.659). Also, the mean total knowledge scores (*P =* 0.793) had no significant differences between groups.

Most of the participants reported that Egypt announced hMPV-positive cases (327; 46.1%), with the superiority of GPs (109; 50.5%) over the community and clinical pharmacists (*P =* 0.04). However, the three groups gave similar answers about positive cases in neighboring countries such as Saudi Arabia and Emirates (*P =* 0.061).

Table 2 shows that clinical pharmacists reported the highest responses regarding the correct viral incubation period (55.9%; *P =* 0.04). However, responses on disease nature (*P =* 0.135), disease symptoms (*P =* 0.563), identifying different cutaneous lesions (*P =* 0.416), and ways of disease transmission (*P =* 0.104) were comparable between groups.

**Table 2:** Responses on different Disease-related aspects of hMPV per study group

Variables	Community pharmacists	Clinical pharmacists	General practitioners	*P*-value
Disease nature				
Viral	253 (93.0%)	214 (96.4%)	205 (94.9%)	0.135
Bacterial	12 (4.4%)	8 (3.6%)	9 (4.2%)	
Parasitic	7 (2.6%)	0	2 (0.9%)	
Incubation period				
5–13 and up to 21 days	127 (46.7%)	124 (55.9%)	111 (51.4%)	0.043
More or less	24 (8.8%)	18 (8.1%)	7 (3.2%)	
Unable to identify	121 (44.5%)	80 (36.0%)	98 (45.4%)	
Disease symptoms				
Lymphadenopathy	210 (77.2%)	176 (79.3%)	171 (79.2%)	0.563
Fever	144 (52.9%)	111 (50%)	104 (48.1%)	
Flu-like symptoms	215 (79.0%)	180 (81.1%)	175 (81.0%)	
Smallpox like symptoms	228 (83.8%)	190 (85.6%)	184 (85.2%)	
Identify skin lesions				
Rash	133 (48.9%)	118 (53.2%)	96 (44.4%)	0.416
Papule	83 (30.5%)	70 (31.5%)	79 (36.6%)	
Vesicle	125 (45.9%)	98 (44.1%)	116 (53.7%)	
Pustule	22 (8.1%)	19 (8.6%)	32 (14.8%)	
Unable to identify	65 (23.9%)	40 (18.0%)	41 (18.9%)	
Disease transmission				
Direct contact with a patient	211 (77.6%)	149 (67.1%)	166 (76.9%)	0.104
Contact with infected body fluids	91 (33.5%)	79 (35.6%)	88 (40.7%)	
Sexual transmission	76 (28.0%)	71 (32.0%)	71 (32.9%)	
Airborne/droplets	88 (32.4%)	81 (36.5%)	60 (27.8%)	

Statistically significant (*p* < 0.05)

Data are reported as numbers (percentages)

Moreover, 23.9% of community pharmacists, 18.0% of clinical pharmacists, and 18.9% of GPs failed to recognize any hMPV skin lesions.

**Fig. 2:**
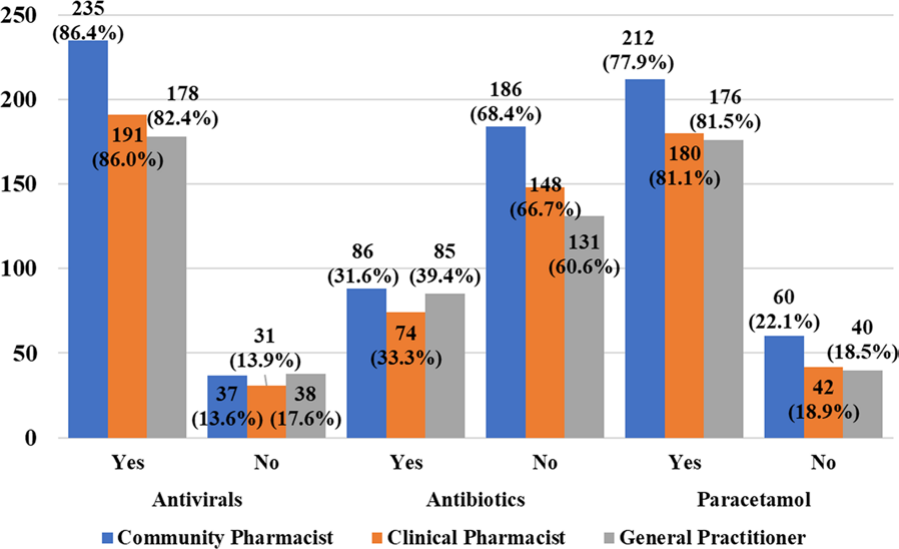
Responses on Disease Management per study groups. *P-value between groups = 0.725 for antivirals, 0.253 for antibiotics, and 0.803 for paracetamol

From Fig. 2, responses identifying the usefulness of antivirals (86.4%) and excluding antibiotics (68.4%) from the management of hMPV were similar between community and clinical pharmacists and GPs.

**Fig. 3:**
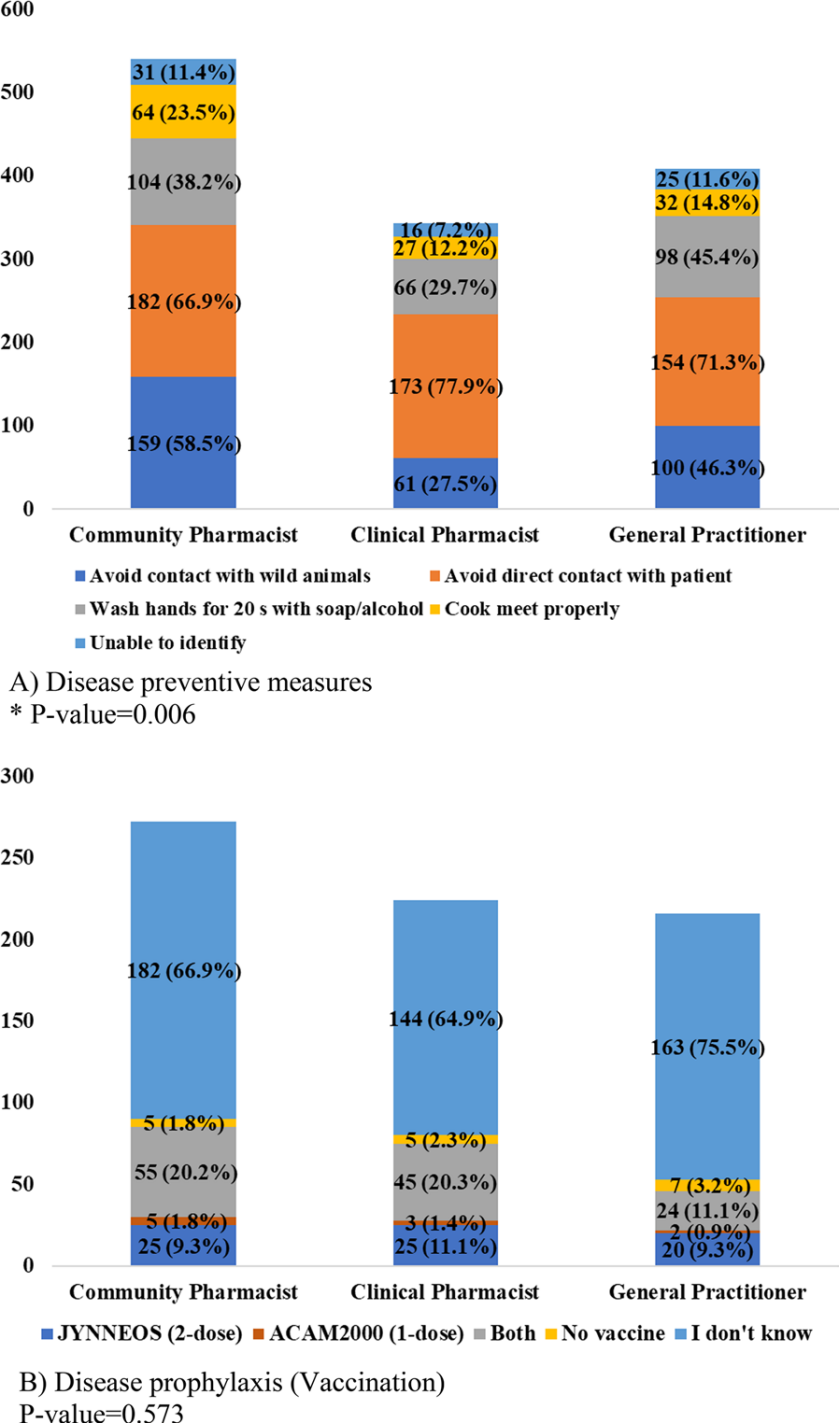
Responses on disease prevention and Vaccination per study group

Community pharmacists were the best to mention different aspects of Preventive measures (*P =* 0.006). Also, clinical pharmacists were the lowest group (7.2%) to report failing to recognize any preventive measures for hMPV transmission to humans (Fig. 3a).

According to CDC reports, 11.1% of Clinical pharmacists versus 9.3% of community pharmacists and GPs successfully reported JYNNEOS (2-dose) for smallpox as the recommended vaccine. 75.5% of GPs vs. 69.9% of the community and 65.9% of clinical pharmacists failed to recognize the recommended vaccine from the two mentioned vaccines. At the same time, all groups minimally reported no vaccine available for hMPV (Fig. 3).

The Knowledge Scores were calculated for each participant based on questionnaire items and categorized into three main domains (disease-related aspects, prevention and prophylaxis, and management). Each good answer was given a score of one and summed up to a total score. The total knowledge scores for all participants ranged from 4 to 24. The following mean ± SD (12.7 ± 3.3) was used as a cut-off value to categorize total knowledge scores in each participant into good knowledge (mean score ≥ 13) or poor knowledge (mean score < 13). Using a cut-off of 13 or more, nearly 358/710 (50.4%) of participants presented a good knowledge about hMPV (community pharmacists; 137 (50.4%), clinical pharmacists; 117 (52.7%), and GPs; 104 (48.1%) with *P* value = 0.826). After that, we calculated % of respondents in each questionnaire domain separately (See Table 3).

**Table 3:** Total knowledge scores and subdomain scores

		Community pharmacist	Clinical pharmacist	General practitioner	*P*-value
Total Knowledge scores	Range	4–23	5–24	4–23	
	Mean ± SD	12.67 ± 3.48	12.86 ± 3.22	12.68 ± 3.20	
	Good	176 (64.7%)	146 (65.8%)	136 (63%)	0.793
	Poor	96 (35.3%)	76 (39.2%)	80 (37%)	
Disease clinical aspects	Good	161 (59.2%)	134 (60.4%)	138 (63.9%)	0.689
	Poor	111 (40.8%)	88 (39.6%)	78 (36.1%)	
Prevention and prophylaxis	Good	72 (26.5%)^#^	37 (16.7%)	35 (16.2%) ^#^	0.048
	Poor	200 (73.5%)	185 (83.3%)	181 (83.8%)	
Management	Good	242 (89%)	198 (89.2%)	192 (89%)	0.324
	Poor	30 (11%)	24 (10.8%)	24 (11%)	

^#^*P* value = 0.037

In Table 4, A correlation between getting medical education about hMPV and different questionnaire items was assessed. Medical education was related to high knowledge scores in the three groups (community pharmacists (*r* = 0.205; *P* = 0.001), clinical pharmacists (*r* = 0.315; *P* < 0.0001), and GPs (*r* = 0.229; *P =* 0.001)).

**Table 4:** Relationship between Medical Education and Different questionnaire items

Medical education Variables	Community pharmacist		Clinical pharmacist		General practitioner	
	*r*	*P* value	*r*	*P* value	*r*	*P* value
Total knowledge scores	0.205	0.001	0.315	0.0001	0.229	0.001
Management scores	− 0.070	0.250	0.028	0.678	0.083	0.222
Prophylaxis and prevention scores	0.098	0.108	0.207	0.002	0.156	0.022
Disease-related aspects scores	0.204	0.001	0.279	0.0001	0.150	0.028
Age	0.051	0.399	− 0.019	0.775	0.140	0.040
Gender	0.054	0.376	− 0.125	0.062	0.147	0.030
Residence	− 0.022	0.715	0.065	0.335	0.020	0.768

Statistically significant (*p* < 0.05)

In the community pharmacy group, medical education was associated with increased knowledge about disease aspects (disease nature, statistics, and incubation period) (*r* = 0.204; *P =* 0.001).

In the clinical pharmacy group, medical education was associated with a raised knowledge about different disease aspects (disease nature, statistics, and incubation period) (*r* = 0.279; *P* < 0.0001) and disease prevention, prophylaxis, and management (*r* = 0.207; *P =* 0.002).

In the GPs group, medical education was associated with a raised knowledge about different disease aspects (disease nature, statistics, and incubation period) (*r* = 0.150; *P =* 0.02), age (*r* = 0.140; *P =* 0.04), gender (*r* = 0.147; *P =* 0.03), and disease prevention, prophylaxis, and management (*r* = 0.156; *P =* 0.02).

## Discussion

Until May 2022, hMPV was considered a neglected infectious disease. The continued growth of hMPV places an undue burden on HCPs [14]. WHO declared hMPV a major public health emergency due to its rapid spread among non-endemic countries [15].

Being an international concern, a collaborative response of all HCPs and the government is necessary for early screening, detection, applying preventive measures, and managing cases. However, among the challenges faced in this outbreak is the lack of knowledge about hMPV [16].

During the COVID-19 crisis, Pharmacists were described as key players and one of the most crucial HCPs providing medical care and awareness. Previous studies that described medical practices in Egypt placed community pharmacists as the closest HCPs to the public [11, 12].

With the current challenging situation, we need pharmacists and GPs to collaborate to identify suspected cases rapidly, promote public awareness, and manage patients [8]. Aside from strengthening governmental surveillance tools, creating an effective response to this outbreak necessitates increasing HCP's Knowledge and confidence [16]. Therefore, the current study aimed to assess the Knowledge of pharmacists and GPs (frontline HCPs) and strengthen their awareness of hMPV disease.

According to the CDC, in October 2022, 254 cases were discovered in Israel, 17 in Sudan, 1 in Jordan, 8 in KSA, and 11 in Lebanon [3]. The previous countries shared boundaries with Egypt. As a re-emerging disease, hMPV is uncommon in the Middle Eastern region. Moreover, nearly 40% of all groups did not hear about positive cases in Egypt or nearby countries.

Across the study, the participants presented an acceptable, accurate, and similar knowledge of hMPV. The current data indicates that merely 50% of each study group could answer the questionnaire items successfully. Clinical and community pharmacists presented similar knowledge to that of the GPs. This level of knowledge is acceptable as it is unusual for our participants to encounter such cases; however, dealing with cases is crucial for enhancing HCP knowledge [17].

Evaluating the knowledge of pharmacists and physicians would help to set measures to manage, prevent, and control the hMPV outbreak. It is vital to highlight the role of pharmacists and GPs’ Knowledge in educating the public and minimizing misinformation accompanied by infectious disease outbreaks [9, 18].

During the past COVID-19 pandemic, the viral spread of wrong information, especially through social media, amplified conspiratorial ideas and negatively impacted social, psychological, and health-related aspects [19, 20]. The demonstrated high Knowledge would be translated into providing corrective information and promoting a rapid and vigilant response.

The past results encouraged the authors to provide valid answers with references after completing the survey to raise pharmacists' and GPs' Knowledge about the current outbreak.

The current results are satisfactory, considering previous studies reporting low Knowledge among GPs and other HCPs [8, 14]. Also, previous studies reported significant knowledge gaps between HCPs [7, 8, 14]. As anticipated, studies on HCPs showed higher knowledge levels than the general population and university students [9, 16]. All studies on HCPs focused on estimating the knowledge of physicians, nurses, or allied health professionals. This study is the first to focus on the knowledge of hMPV among the community and clinical pharmacists.

Previous studies on HCP highlighted the presence of significant variations in knowledge about hMPV among HCPs, favouring physicians over allied health professionals (*P* < 0.03). However, nurses were more confident in diagnosing and managing this disease [7]. Also, a study in Jordan agreed that physicians showed a higher knowledge level of hMPV [9]. The current results indicated insignificant differences in the knowledge scores between pharmacists and GPs. However, clinical pharmacists reported higher mean knowledge scores than community pharmacists and GPs.

After five to 21 days of viral exposure, symptoms begin to appear [21]. Clinical pharmacists outperformed community pharmacists and GPs in providing the correct answers about the right incubation period.

The clinical picture of hMPV has been inconsistent in confirmed cases in the current outbreak [22]. Many patients are not experiencing the common symptoms of hMPV, including oral/vaginal/peri-anal lesions, fever, lymphadenopathy, cutaneous symptoms, and swallowing difficulties [23].

This disease shares a similar clinical picture with smallpox, with mild symptoms and a better prognosis [24]. The transmission of hMPV occurs via direct contact with infected animals, humans, and body fluids. Face-to-face transmission is very likely, but airborne transmission has not been reported yet [25].

Clinical pharmacists, community pharmacists, and GPs provided similar answers to the questions about the nature of the disease, non-cutaneous manifestations, and ways of transmission; this reflects a good knowledge about the disease's nature with responses > 90% in each group and fairly answered the proper incubation period (~ 50% correct answers in each group).

A low level of response across all study groups (less than 40%) corresponds to a gap in knowledge about hMPV cutaneous symptoms other than skin rashes and disease transmission other than direct contact with patients.

Skin lesions are the most noticeable sign (rashes, papules, vesicles, or pustules), which begin in the face and extend to the whole body in severe cases [26]. This finding may correlate to defects in case diagnosis, which correspond to ordering unnecessary laboratory tests and wasting governmental resources [27]. Earlier studies showed gaps in knowledge about non-cutaneous symptoms and human-to-human transmission [7, 9, 28].

Pharmacists have an increasing role in implementing preventive measures during emergent outbreaks. Moreover, community pharmacists administered COVID-19 and provided guidance and education to the public with satisfactory levels of confidence in many Arab countries [29].

However, another gap identified in this research is the knowledge about ways of transmission, prevention, and prophylaxis. From the previous results, it is very noticeable that all three groups have poor knowledge about vaccination available and other preventive measures; however, there was a statistically significant difference between community pharmacists and GPs.

These complement the results of a previous study, which highlighted the need to raise HCPs’ Knowledge about ways of transmission and subsequently implement proper disease control measures [7].

hMPV is a self-limiting disease that requires symptomatic treatment as antipyretics. Antibiotics are not recommended unless a secondary bacterial infection is a complication. Based on animal and human research, the European Medicines Agency (EMA) authorized tecovirimat, an antiviral drug licensed for smallpox, for hMPV in 2022 [30].

Smallpox vaccines are known to have high protection against hMPV. New vaccines are available, but neither the drugs nor the vaccines are freely available on the market  [23]. According to CDC recommendations, JYNNEOS (2-dose) for smallpox is the preferred vaccine for hMPV [3].

Most community pharmacists reported higher knowledge scores in questions related to disease preventive measures (merely 50%), which may correlate to the acceptable raising of infection control culture among the public.

However, community and clinical pharmacists and GPs reported comparable disease management and vaccination, which may correlate to similar confidence levels and skills in managing hMPV cases.

Unfortunately, 30% or more participants across study groups answered that antibiotics were among the treatment options for hMPV. Egypt and the Middle East suffer from antibiotic resistance; also, in the COVID-19 era, the consumption of antibiotics without clinical indication reaches its maximum [11, 31]. This particular issue needs a strict warning to HCPs and the public to avoid aggravating this problem.

By identifying the previously mentioned knowledge gaps, our main aim was to guide the Egyptian MOHP and other health policymakers to provide well-structured, evidence-based, and tailored training programs addressed to pharmacists and GPs separately about prevention and management to minimize the spreading of hMPV and other re-emerging infectious diseases.

After investigating the correlation between medical education and different questionnaire items among study groups, expectedly, medical education was positively correlated with high total knowledge scores among all participants.

Furthermore, medical education was associated with a raised knowledge about different disease aspects (disease nature, statistics, and incubation period) across all study groups and disease prevention, prophylaxis, and management for clinical pharmacists and GPs. Finally, medical education was associated with older age and females for GPs. On the contrary, a study on physicians found that younger participants had higher knowledge and was explained as better access to the Internet at a younger age [8, 32].

The previous result may be explained as older GPs would link to higher rates of acquiring training workshops and scientific conferences, which will be translated into higher knowledge and skills. As well as, older GPs are capable of consuming guidelines and have higher expertise in clinical practice [33].

The study was limited by the cross-sectional design, which limits causal associations. In such study design, recall and selection biases are inevitable.

## Conclusion

Pharmacists and GPs presented similar reasonable knowledge scores about managing and diagnosing hMPV infection. Community pharmacists were more aware of preventive measures for this infectious disease. This acceptable knowledge status in pharmacists and GPs would reflect a satisfactory confidence level in managing infected cases; however, gaps remain. Tailored disease prevention and vaccination training is required to raise their knowledge and confidence levels and ensure optimal patient care.

## Data Availability

The datasets used and/or analyzed during the current study are available from the corresponding author on a reasonable request.
